# ﻿*Argopistes* Motschulsky from Madagascar with descriptions of six new species (Coleoptera, Chrysomelidae, Galerucinae, Alticini)

**DOI:** 10.3897/zookeys.1202.122977

**Published:** 2024-05-27

**Authors:** Maurizio Biondi, Mattia Iannella, Paola D'Alessandro

**Affiliations:** 1 Department of Life, Health and Environmental Sciences, University of L’Aquila, 67100 L’Aquila, Italy University of L’Aquila L’Aquila Italy

**Keywords:** Afrotropical region, Ecological Niche Modelling, flea beetles, new species, synonymy

## Abstract

The revision of the flea beetle genus *Argopistes* Motschulsky, 1860 in Madagascar is provided. Six new species are described: *Argopistesjanakmoravecorum***sp. nov.**, *A.laterosinuatus***sp. nov.**, and *A.vadoni* from the northern area; *A.jenisi***sp. nov.**, *A.keiseri***sp. nov.**, and *A.seyrigi***sp. nov.** from the central area. A new synonym of *Argopistesbrunneus* Weise, 1895 is established: *A.sexguttatus* Weise, 1895, **syn. nov.**, since *A.sexguttatus* is shown to be a chromatic form of *A.brunneus*. A diagnostic key of the seven Malagasy *Argopistes* species is provided, with photographs of the habitus, median lobe of the aedeagus, and spermatheca. Finally, based on known occurrences, the current suitable areas for this flea beetle genus in Madagascar are estimated using Ecological Niche Modelling (ENM) techniques.

## ﻿Introduction

Madagascar is considered one of the world’s most important biodiversity hotspots thanks to the many field campaigns conducted since the 17^th^ century that documented its species richness (e.g., Andriamialisoa and Langrand 2022). Nevertheless, its faunistic diversity is still only partially known, especially for some invertebrate groups, including flea beetles (Coleoptera, Chrysomelidae, Galerucinae, Alticini) (Iannella et al. 2019). Biondi and D’Alessandro (2012) reported approximately 260 flea beetle species in 39 genera from Madagascar, of which 13 were endemics. Subsequent published papers and additional material preserved in public and private collections demonstrate that these numbers are significantly underestimated (Biondi and D’Alessandro 2013a, 2013b, 2016; D’Alessandro et al. 2014).

The flea beetle genus *Argopistes* Motschulsky, 1860 was described based on a new species from Siberia, *A.biplagiatus* Motschulsky, 1860, the type species by monotypy. The genus was subsequently reported for the Afrotropical, Australian, Neotropical, Oriental, and Palearctic Regions, with a total of 38 species (Blanco and Konstantinov 2013).

Nadein (2015), through a morphological-based cladistic analysis, attributed *Argopistes* to the subtribe Diboliina with *Dibolia* Latreille, *Megistops* Boheman, and *Paradibolia* Baly. More recent papers based on the use of molecular data considered *Argopistes* closely related to *Apteropeda* Chevrolat (Hlaka et al. 2022), *Dibolia*, and *Apteropeda* (Letsch and Beran 2023), or *Dibolia* and *Sphaeroderma* Stephens (Douglas et al. 2023). Nevertheless, all authors agreed that additional molecular data and denser taxon sampling are required to provide a robust basis for establishing internal relationships among Alticini and possible subtribal classifications.

Blanco and Konstantinov (2013) recognized nine valid *Argopistes* species in the Afrotropical Region, including Madagascar, but at least 13 new species are currently being described from that area, based on new unpublished material (M. Biondi, unpublished data). In this paper, we provided a revision of the Malagasy *Argopistes* species. We described *Argopistesjanakmoravecorum* sp. nov., *A.laterosinuatus* sp. nov., and *A.vadoni* sp. nov. from the northern area, and *A.jenisi* sp. nov., *A.keiseri* sp. nov., and *A.seyrigi* sp. nov. from the central area. We proposed a new synonym of *Argopistesbrunneus* Weise, 1895, *A.sexguttatus* Weise, 1895, syn. nov. Finally, based on the known occurrences, we reconstructed the current high suitability areas for the *Argopistes* species in Madagascar using Ecological Niche Modelling (ENM) techniques.

## ﻿Materials and methods

Material examined consisted of dried pinned specimens preserved in the institutions listed in the Abbreviations section. Specimens were examined, measured, and dissected using a Leica M205C stereomicroscope. Photographs were taken using a Leica DMC5400 camera and compiled with the focus stacking technique using Zerene Stacker software v. 1.04. Scanning electron micrographs were taken using a Hitachi TM-1000. Terminology followed Schmitt et al. (2023) for the median lobe of the aedeagus, and Döberl (1986) and Suzuki (1988) for the spermatheca. Geographic coordinates were reported in the Degrees and Decimal Minutes format (DDM) using the WGS84 datum; information included in square brackets was added by the authors, using the Google Earth website for coordinates and geographic information. Chorotypes follow Biondi and D’Alessandro (2006). Vegetation division names refer to Sayre et al. (2013). Abbreviations for the depositories followed the list on the website The Insect and Spider Collections of the World (Evenhuis 2023). Exact label data were cited for all type specimens of the examined or described species; a double slash (//) divided the data on different labels and a single slash (/) divided the data in different rows.

Ecological Niche Models (ENMs) were built based on all the known occurrences, and on 19 temperature- and precipitation-related “bioclimatic” raster variables selected as candidate predictors from the Worldclim.org repository (Fick and Hijmans 2017), namely **BIO1**: annual mean temperature, **BIO2**: mean diurnal range (mean of monthly (max temp–min temp)), **BIO3**: isothermality (BIO2/BIO7) (×100), **BIO4**: temperature seasonality (standard deviation ×100), **BIO5**: max temperature of warmest month, **BIO6**: min temperature of coldest month, **BIO7**: temperature annual range (BIO5-BIO6), **BIO8**: mean temperature of wettest quarter, BIO9: mean temperature of driest quarter, **BIO10**: mean temperature of warmest quarter, **BIO11**: mean temperature of coldest quarter, **BIO12**: annual precipitation, **BIO13**: precipitation of wettest month, **BIO14**: precipitation of driest month, **BIO15**: precipitation seasonality (coefficient of variation), **BIO16**: precipitation of wettest quarter, **BIO17**: precipitation of driest quarter, **BIO18**: precipitation of warmest quarter, and **BIO19**: precipitation of coldest quarter. To avoid potential correlation among variables, which leads to the lowering of the model’s performance, we measured both the variance inflation factor (VIF), setting the threshold = 10 (Guisan et al. 2017), and Pearson’s *r* (|*r*| < 0.75, following Dormann (2007) and Elith et al. (2006)); for this purpose, we used the ‘vifstep’ and ‘vifcor’ functions of the ‘usdm’ R package (Naimi 2017). The variables obtained as the analyses’ outcomes were then selected as predictors to calibrate the models. The ENMs were performed using the “Presence-only Prediction (Maxent)” tool in the ArcGis Spatial Analyst. This tool permits to infer, based on a set of environmental predictors and occurrence localities (specifically, a presence-only dataset), the suitability of a certain taxon across an area, also giving marginal response curves of the predictors with respect to the predicted suitability. Its main advantage in terms of model discrimination capability is the possibility to calibrate and evaluate performances through a spatial jackknifing procedure (ESRI, 2023). Moreover, the ENM’s performance was evaluated by both assessing the Area Under the Curve (AUC) of the ROC (Phillips et al. 2006), automatically resulting from the ArcGIS tool, and the Continuous Boyce Index (CBI), particularly useful for presence-only models (Hirzel et al, 2006; Leroy et al. 2018), calculated through the ‘ecospat.boyce’ function in the ‘ecospat’ R package (Di Cola et al. 2017).

### ﻿Abbreviations

#### ﻿Collections and repositories

**BAQ** Italy, University of L’Aquila, Collection of M. Biondi;

**NHMB**Switzerland, Basel, Naturhistorisches Museum;

**RMCA** Belgium, Tervuren, Musée Royal de l’Afrique Centrale;

**ZMHB**Germany, Berlin, Museum für Naturkunde.

#### ﻿Biometrics

**LA** numerical sequence from base to apex of each antennomere, proportional to the length of the first antennomere;

**LAED** length of median lobe of the aedeagus;

**LAN** length of antennae;

**LB** total body length (from apical margin of head to apex of elytra);

**LE** maximum length of elytra;

**LF** maximum length of hind femora;

**LP** medial length of pronotum;

**LSPC** maximum length of spermathecal capsule;

**WE** maximum width of elytra combined;

**WF** maximum width of hind femora;

**WP** maximum width of pronotum.

## ﻿Results

### ﻿Taxonomy

#### ﻿ Argopistes brunneus

Taxon classificationAnimaliaColeopteraChrysomelidae

Weise

BC47E641-3FAA-5781-B5DA-8F34F389DE12

Argopistes brunneus Weise, 1895: 336.
Argopistes sexguttatus Weise, 1895: 336. syn. nov.

##### Type material examined.

***Holotype*** of *Argopistesbrunneus* ♂: “Madagasc. / Pipitz // Madagasc / 195 / Pipitz” [Madagascar, Dr. Pipitz leg.] [handwritten on light blue cards] “*Argopistes* / *brunneus* / m” [handwritten on white card], “HOLOTYPUS / *Argopistesbrunneus* Weise / labelled by MNHUB” [printed on red card], (ZMHB).

***Holotype*** of *Argopistessexguttatus* ♂: “Madagasc. / Pipitz // Madagasc / 193 / Pipitz” [Madagascar, Dr. Pipitz leg.] [handwritten on light blue cards] “*Argopistes* / *6-guttatus*/ m” [handwritten on white card]”, “HOLOTYPUS / *Argopistessexguttatus* Weise / labelled by MNHUB” [printed on red card], (ZMHB).

##### Additional material examined.

1 spec., Madagascar Nord, Antsiranana prov., Amber Gebirge [~12°2.20'S, 49°15.02'E] (ZMHB); 1 spec., Madagascar, Toamasina prov., forêt de Fito, ex Coll. Dr. Breuning, [17°59.99'S, 48°50.50'E] (RMCA); 2 specs, Madagascar, Tamatave [= Toamasina, 18°8.97'S, 49°24.14'E] (ZMHB); 2 specs, ibid, Coll. Clavareau (RMCA); 1 spec., Madagascar, Fianarantsoa prov., Ranomafana env. [21°15.76'S, 47°27.12'E], 28.i–6.ii.1995, Ivo Jeniš leg. (BAQ).

##### Redescription.

**Figure 1.**
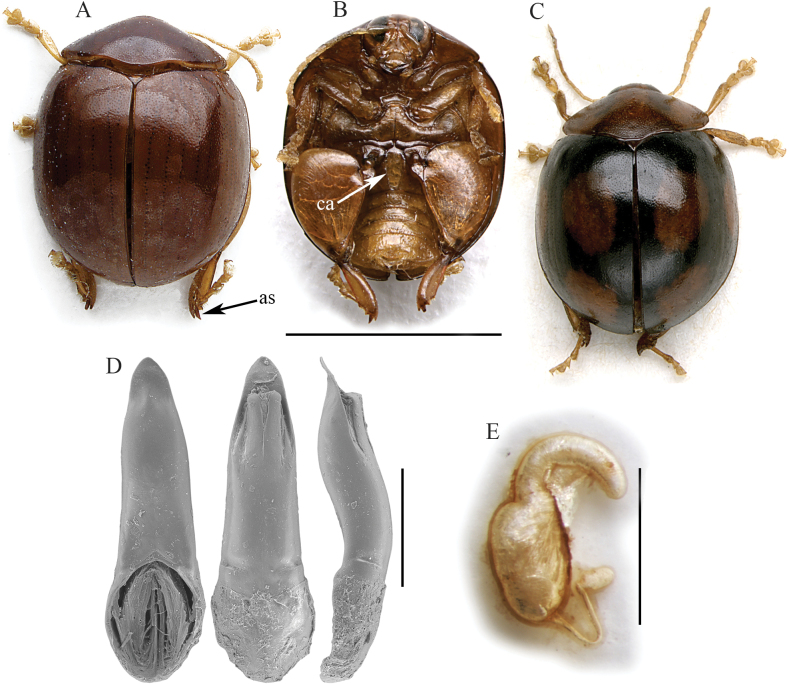
*Argopistesbrunneus* Weise. **A** holotype of *A.brunneus*, habitus in dorsal view **B** ibid, ventral view **C** holotype of *A.sexguttatus* Weise, habitus in dorsal view **D** median lobe of the aedeagus, from left to right in dorsal, ventral, and lateral view, from Tamatave **E** spermatheca, from Tamatave. Abbreviations: as: apical spur of hind tibia; ca: central area of the first abdominal sternite bordered by ridges. Scale bars: 3 mm (**A, B, C**); 500 μm (**D**); 300 μm (**E**).

Body subrounded in dorsal view, with slightly parallel sides (Fig. 1A, C), strongly convex in lateral view; total length of body (LB) = 3.82 ± 0.13 mm (3.68 ≤ LB ≤ 3.98 mm) in male, and 3.79 ± 0.06 mm (3.72 ≤ LB ≤ 3.84 mm) in female; maximum pronotal width at the base: WP = 2.33 ± 0.05 mm (2.28 ≤ WP ≤ 2.40 mm) in male, and 2.27 ± 0.07 mm (2.20 ≤ WP ≤ 2.36 mm) in female; maximum width of elytra in the middle: WE = 3.15 ± 0.07 mm (3.08 ≤ WE ≤ 3.24 mm) in male, and WE = 3.19 ± 0.10 mm (3.04 ≤ WE ≤ 3.24 mm) in female; WE/WP = 1.35 ± 0.03 (1.32 ≤ WE/WP ≤ 1.40) in male, and WE/WP = 1.41 ± 0.04 (1.36 ≤ WE/WP ≤ 1.45) in female.

Color of the dorsal integument variable (Fig. 1A, C): entirely black; entirely brown; with black elytral disc blending in reddish brown towards the pronotum and the elytral margins; with brown head and pronotum, and black elytra with brown patches; antennae (Fig. 1B) yellowish; hind legs brown or paler; fore- and middle legs generally yellowish (Fig. 1B); ventral parts mostly brownish (Fig. 1B).

Head entirely hidden by the pronotum; vertex with very small, irregular punctation and a pair of large setiferous pores; frontal calli barely delimited, not raised; frons moderately elongate, its surface irregular, roughly wrinkled; frontal ridge elongate, thin and sharp; frontogenal sutures distinctly raised; eyes large, elongate, slightly kidney-shaped; interantennal space clearly narrower than antennal sockets. Antennae (Fig. 1B) filiform, as long as ~ 1/2 the body length: LAN = 1.98 ± 0.08 mm (1.88 ≤ LAN ≤ 2.08 mm) in male, and 1.71 ± 0.08 mm (1.64 ≤ LAN ≤ 1.80 mm) in female, and LAN/LB = 0.52 ± 0.01 (0.51 ≤ LAN/LB ≤ 0.53) in male, and 0.45 ± 0.02 (0.43 ≤ LAN/LB ≤ 0.47) in female; segments 1–2 thicker; segments 3–11 slightly and gradually flattened.

Pronotum (Fig. 1A, C) distinctly transverse: LP = 1.06 ± 0.06 mm (1.00 ≤ LP ≤ 1.12 mm) in male, and 1.05 ± 0.04 mm (1.00 ≤ LP ≤ 1.08 mm) in female, and WP/LP = 2.20 ± 0.07 (2.14 ≤ WP/LP ≤ 2.28) in male, and 2.16 ± 0.05 (2.09 ≤ WP/LP ≤ 2.20) in female; lateral margins strongly convergent anteriorly, weakly curved to straight, very weakly expanded, not visible in dorsal view; basal margin arcuate and moderately sinuate; surface smooth, sparsely micropunctate, with very dense, small punctation; surface barely raised parallel to the lateral margins, near the posterior angles; a large setiferous pore at the anterior angles. Scutellum small, subtriangular.

Elytra (Fig. 1A, C) distinctly curved but slightly parallel in the middle third, moderately longer than wide, jointly rounded apically; lateral margins finely bordered, visible in dorsal view; surface smooth to sparsely micropunctate; main punctation mostly confused, very dense, small, slightly shallower than on pronotum but more impressed laterally; 9 (+ 1 sutural) regular lines are visible in paler specimens due to the blackened punctures (Fig. 1A); LE = 3.38 ± 0.10 mm (3.24 ≤ LE ≤ 3.50 mm) in male, and 3.43 ± 0.09 mm (3.32 ≤ LE ≤ 3.52 mm) in female; WE/LE = 0.93 ± 0.2 (0.91 ≤ WE/LE ≤ 0.95) in male, and 0.93 ± 0.03 (0.89 ≤ WE/LE ≤ 0.98) in female; LE/LP = 3.19 ± 0.09 (3.09 ≤ LE/LP ≤ 3.32) in male, and 3.27 ± 0.16 (3.07 ≤ LE/LP ≤ 3.40) in female. Humeral calli moderately raised. Macropterous.

Prosternum with posteriorly open procoxal cavities and large intercoxal prosternal process. Mesosternum very short. First abdominal sternite approx. as long as fifth (Fig. 1B); its central area bordered by ridges is quite narrow and subrhomboidal. Anterior and middle legs without modifications. Posterior femora greatly swollen (WF/LF = 0.68 ± 0.01), elongate-subtriangular; posterior tibiae thick, distinctly shorter than femora, apically widened and prolonged into a spur-like process on inner side; outer side of hind tibia apically toothed; apical spur of hind tibiae simple, lanceolate; first metatarsomere moderately enlarged in male.

Median lobe of the aedeagus (Fig. 1D) with smooth surface; in ventral view is tapered towards the apex, slightly sinuate laterally; in lateral view moderately curved, with sinuate ventral outline and straight apex; dorsal ligula formed by a medially incised central lobe, and two thinner lateral lobes; its base at apical ~ 1/3; LAED = 1.41 ± 0.04 mm (1.36 ≤ LAED ≤ 1.48 mm); LE/LAED = 2.40 ± 0.08 (2.30 ≤ LE/LAED ≤ 2.50).

Basal part of the spermatheca (Fig. 1E) subcylindrical, dorsally enlarged; distal part curved, elongate, uniform in thickness, with collum generally not distinguishable from the apical part; ductus subapically inserted and oriented, thin, quite short, uncoiled; LSPC = 0.38 ± 0.01 mm (0.36 ≤ LSPC ≤ 0.38 mm); LE/LSPC = 9.14 ± 0.39 (8.74 ≤ LE/LSPC ≤ 9.61).

##### Remarks.

*Argopistesbrunneus* is distinguishable from the other Malagasy *Argopistes* species by the slightly parallel sides in dorsal view (Fig. 1A, C), and the first abdominal sternite, whose central surface bordered by ridges is narrow and convergent posteriorly (Fig. 1B). The median lobe of the aedeagus and spermatheca (Fig. 1D, E) are also diagnostic. *Argopistessexguttatus* Weise is here synonymized with *A.brunneus*, simply representing one of its chromatic forms.

##### Distribution.

Northern, eastern, and central Madagascar (Antsiranana, Toamasina, and Fianarantsoa provinces; Fig. 8A). Malagasy chorotype.

##### Ecological notes.

Host plant unknown. Collection localities fall within areas characterized by the vegetation divisions of ‘Malagasy Evergreen & Semi-Evergreen Forest’ and ‘Malagasy Dry Deciduous & Evergreen Forest & Woodland’.

#### ﻿ Argopistes janakmoravecorum sp. nov.

Taxon classificationAnimaliaColeopteraChrysomelidae

80F3E295-820C-515A-A8CA-13AC9E3238B4

https://zoobank.org/62088B6A-315D-4294-93DD-237DAC0E4BDE

Figs 2 , 8A

##### Type material.

***Holotype*** ♀: “Madagascar Nord / 800-1000 m / 5 km à est d’Andapa / Lembonibona (1265 m) // forêt degradee, arbres, arbustes / 2.3.1996 / J. Janák + P. Moravec lgt.” [printed on white card] [14°40.63'S; 49°41.63'E] (BAQ).

##### Diagnosis.

**Figure 2.**
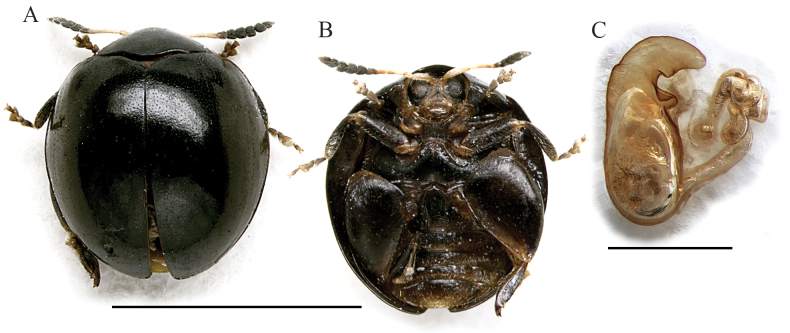
*Argopistesjanakmoravecorum* sp. nov. **A** holotype, habitus in dorsal view **B** ibid, ventral view **C** ibid, spermatheca. Scale bars: 3 mm (**A, B**); 300 μm (**C**).

*Argopistesjanakmoravecorum* sp. nov. is easily distinguishable from the other Afrotropical *Argopistes* species by the combination of black dorsal integuments and clavate antennae with segments 1–5 yellowish and 6–11 blackened (Fig. 2B). Spermatheca is also strongly diagnostic, due to the elongate and distally coiled ductus (Fig. 2C).

##### Description of the holotype

(♀) . Body roundish in dorsal view (Fig. 2A), very convex in lateral view; total length of the body (LB) = 3.00 mm; maximum pronotal width at the base (WP = 1.68 mm); maximum width of elytra in the middle (WE = 2.56 mm); WE/WP = 1.52. Dorsal integuments black with weak metallic reflections (Fig. 2A); scutellum brownish; head brownish; frons and mouthparts yellowish; antennae (Fig. 2B) with segments 1–5 yellowish, 6 dark brown, 7–10 black, 11 black but distally paler; ventral parts (Fig. 2B) mostly dark brown; legs with femora and tibiae blackish, tarsi partially dark brown and articulations yellowish (Fig. 2B). Head entirely hidden by the pronotum; vertex irregularly punctate, with a pair of large setiferous pores; frontal calli barely delimited, not raised; frons elongate, with rough, irregular surface; frontal ridge barely detectable; frontogenal sutures weakly raised; eyes large, elongate, slightly kidney-shaped; interantennal space clearly narrower than antennal sockets. Antennae (Fig. 2B) clavate, as long as ~ 1/2 the body length (LAN = 1.36 mm; LAN/LB = 0.45); LA = 100:46:32:53:58:42:47:54:49:42:69. Pronotum (Fig. 2A) clearly transverse (LP = 0.78 mm; WP/LP = 2.15); lateral margins strongly convergent anteriorly and slightly folded ventrally, moderately curved, barely expanded, not visible in dorsal view; basal margin arcuate and distinctly sinuate; surface finely microreticulate, with very small and dense punctation; surface moderately raised parallel to the lateral margins; a large setiferous pore at the anterior angles. Scutellum small, subtriangular. Elytra (Fig. 2A) (LE: 2.72 mm, LE/LP = 3.49) strongly curved laterally, slightly longer than wide (WE/LE = 0.94), jointly rounded apically; lateral margins finely bordered, visible in dorsal view; surface subsmooth, with very small and dense punctation, similar to pronotum, mostly confused but arranged in a couple of lines of slightly larger punctures laterally. Humeral calli moderately raised. Macropterous. Prosternum with posteriorly open procoxal cavities and large intercoxal prosternal process. Mesosternum very short. First abdominal sternite slightly longer than fifth (Fig. 2B); its central area bordered by ridges is quite wide, arcuate anteriorly and slightly narrowing posteriorly. Anterior and middle legs without modifications. Posterior femora greatly swollen (WF/LF = 0.61), elongate-subtriangular; posterior tibiae thick, distinctly shorter than femora, apically widened and prolonged into a spur-like process on inner side; outer side of hind tibia apically toothed; apical spur of hind tibiae simple, lanceolate. Basal part of the spermatheca (Fig. 2C) subpyriform, with a distinct ventral protrusion close to the distal part; collum very short; apical part short, narrowing towards the apex; ductus ventrally inserted, thickset, elongate, distally coiled; LSPC = 0.48 mm; LE/LSPC = 5.67.

##### Etymology.

The specific epithet refers to the two collectors of the new species: Jiří Janák and Pavel Moravec from the Czech Republic, both esteemed experts on ColeopteraCarabidae. The name was composed by the union of the two surnames, applying Latin plural genitive.

##### Distribution.

Northern Madagascar (Antsiranana province; Fig. 8A). Malagasy chorotype.

##### Ecological notes.

Host plant unknown. The only known occurrence locality falls within an area characterized by the vegetation division ‘Malagasy Evergreen & Semi-Evergreen Forest’.

#### ﻿ Argopistes jenisi sp. nov.

Taxon classificationAnimaliaColeopteraChrysomelidae

1CEFBBCD-9186-56D0-9860-258DD96F5B88

https://zoobank.org/9E7062ED-CDBE-4325-AB7F-E912391A250C

Figs 3 , 8A

##### Type material.

***Holotype*** ♀: “Madagascar / Tamatave prov. / Ambodinifody / 26.12.1996 / Ivo Jeniš leg.” [printed on white card] [18°53.20'S; 48°3.04'E] (BAQ).

##### Diagnosis.

**Figure 3.**
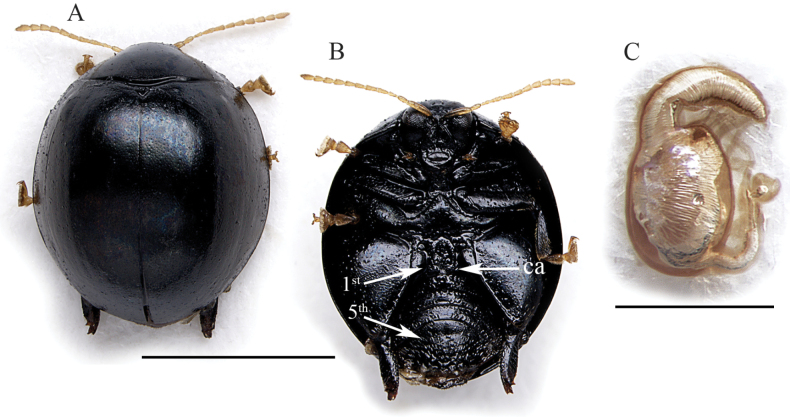
*Argopistesjenisi* sp. nov. **A** holotype, habitus in dorsal view **B** ibid, ventral view **C** ibid, spermatheca. Abbreviations: 1^st^: first abdominal sternite; 5^th^: fifth abdominal sternite; ca: central area of the first abdominal sternite bordered by ridges. Scale bars: 3 mm (**A, B**); 300 μm (**C**).

*Argopistesjenisi* sp. nov. is recognizable by the combination of the following characters: intense black color that contrasts with the yellow antennae, tarsi, and maxillary palpi (Fig. 3A, B); filiform antennae (Fig. 3A, B); wide last abdominal sternite, distinctly longer than first (Fig. 3B). Spermatheca is also diagnostic due to the subglobose basal part ventrally enlarged, and the short ductus, subventrally inserted (Fig. 3C).

##### Description of the holotype

(♀) . Body broadly elliptic in dorsal view (Fig. 3A), strongly convex in lateral view; total length of body (LB) = 4.44 mm; maximum pronotal width at the base (WP = 2.60 mm); maximum width of elytra in the middle (WE = 3.76 mm); WE/WP = 1.45. Dorsal integuments (Fig. 3A) entirely black with evident blueish metallic reflections; ventral parts (Fig. 3B) intensively black; head black; frons and mouthparts black, with yellowish maxillary palpi; antennae entirely yellowish (Fig. 3B); legs, including articulations, black, with yellowish tarsi (Fig. 3B). Head entirely hidden by the pronotum; vertex surface rough and distinctly punctate, with a pair of large setiferous pores; area of frontal calli weakly raised; frons elongate, with rough, irregular surface; frontal ridge thin and short; frontogenal sutures strongly raised; eyes large, elongate, slightly kidney-shaped; interantennal space clearly narrower than antennal sockets. Antennae filiform (Fig. 3B), as long as ~ 1/2 the body length (LAN = 2.12 mm; LAN/LB = 0.48); segments 1–2 thicker; segments 3–11 slightly and gradually flattened; LA = 100:42:33:47:47:40:41:44:43:39:64. Pronotum (Fig. 3A) clearly transverse (LP = 1.20 mm; WP/LP = 2.17); lateral margins strongly convergent anteriorly, weakly curved, weakly expanded, not visible in dorsal view; basal margin arcuate and distinctly sinuate; surface smooth, with very small and very dense punctation; surface moderately raised parallel to the lateral margins; a large setiferous pore at the anterior angles. Scutellum small, subtriangular. Elytra (LE = 4.04 mm; LE/LP = 3.37) distinctly curved laterally (Fig. 3A), distinctly longer than wide (WE/LE = 0.93), jointly rounded apically; lateral margins finely bordered, visible in dorsal view; surface smooth; punctation very small, dense, and confused; points slightly larger towards lateral and apical parts, and partially arranged in some longitudinal lines. Humeral calli moderately raised. Macropterous. Prosternum with posteriorly open procoxal cavities and large intercoxal prosternal process. Mesosternum very short. First abdominal sternite distinctly shorter than fifth (Fig. 3B); its central area bordered by ridges is moderately wide, rounded anteriorly, laterally subparallel. Anterior and middle legs without modifications. Posterior femora greatly swollen (WF/LF = 0.68), elongate-subtriangular; posterior tibiae thick, distinctly shorter than femora, apically widened and prolonged into a spur-like process on inner side; outer side of hind tibia apically toothed; apical spur simple, lanceolate. Spermatheca (LSPC = 0.38 mm; LE/LSPC = 10.63) with apparently wrinkled surface (Fig. 3C); basal part subglobose, with a small protrusion just below the distal part; apical part moderately elongate, narrowing towards the apex; ductus subventrally inserted, quite narrow and short, uncoiled.

##### Etymology.

The specific epithet refers to the collector of the new species: Ivo Jeniš from the Czech Republic, renowned expert on ColeopteraCerambycidae.

##### Distribution.

Central-eastern Madagascar (Toamasina province; Fig. 8A). Malagasy chorotype.

##### Ecological notes.

Host plant unknown. The only known occurrence locality falls within an area characterized by the vegetation division ‘Malagasy Evergreen & Semi-Evergreen Forest’.

#### ﻿ Argopistes keiseri sp. nov.

Taxon classificationAnimaliaColeopteraChrysomelidae

A032F140-3BB7-5883-B1B1-F4D109645B23

https://zoobank.org/D3E13A53-73C9-4BFC-9031-CA836A3E7775

Figs 4 , 8A

##### Type material.

***Holotype*** ♂: “Madagascar / Tamatave prov. / Manankazo env. / 11-12.11.1995 / Ivo Jeniš leg.” [printed on white card] [17°59.26'S; 46°54.20'E] (BAQ). Paratypes. 1 ♂ and 1 ♀ “Madagascar Tam. / Moramanga / 20.xii.1957 F. Keiser” [printed on pink card] // “Non Cocc. Det. H. Fürsch” [printed on white card] [18°56.93'S; 48°13.47'E] (NHMB); 2 ♀♀, “Madagascar / Tamatave prov. / Moramanga env. / 24.2-1.3.1995 / Ivo Jeniš leg.” [printed on white card] [18°56.93'S; 48°13.47'E] (BAQ); 2 ♀♀, “Madagascar / Tamatave prov. / Moramanga env. / 25-27.11.1995 / Ivo Jeniš leg.” [printed on white card] [18°56.93'S; 48°13.47'E] (BAQ); 2 ♀♀, “Madagascar / Tamatave prov. / Moramanga env. / 21-24.12.1996 / Ivo Jeniš leg.” [printed on white card] [18°56.93'S; 48°13.47'E] (BAQ); 1♂ and 1♀, “Madagascar / Tamatave prov. / Maromizaha / 21.II.1995 Ivo Jeniš” [printed on white card] [18°58.57'S; 48°27.90'E] (BAQ); 2♂ and 3♀, “Madagascar / Fianarantsoa prov. / Ranomafana env. / 28.I-6.II.1995 Ivo Jeniš” [printed on white card] [21°15.76'S; 47°27.12'E] (BAQ).

##### Diagnosis.

**Figure 4.**
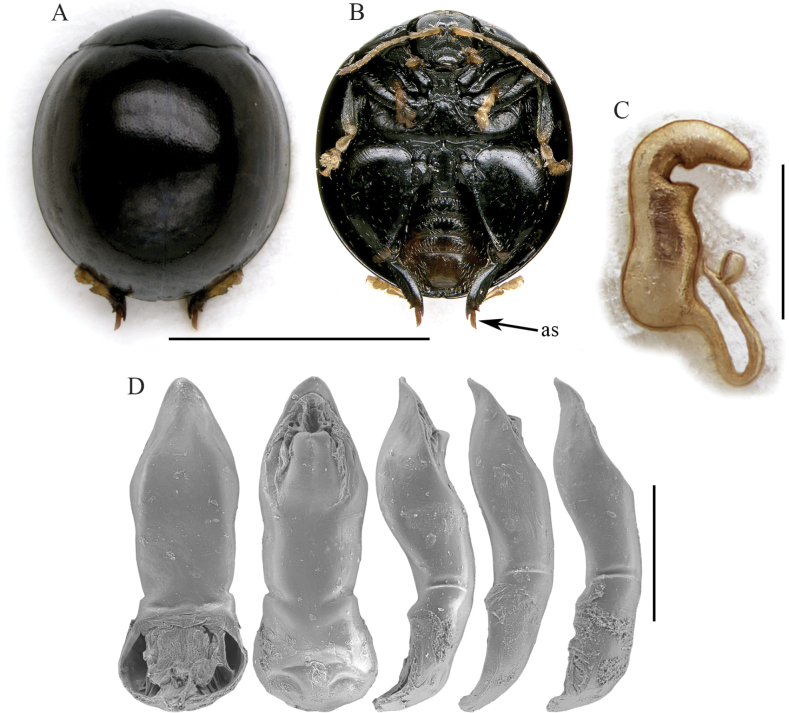
*Argopisteskeiseri* sp. nov. **A** habitus in dorsal view, male from Moramanga **B** ibid, ventral view **C** spermatheca, from Ranomafana **D** median lobe of the aedeagus, from left to right in dorsal, ventral, and lateral view, from Ranomafana, and two additional lateral view from Manankazo, and Maromizama. Abbreviations: as: apical spur of hind tibia. Scale bars: 3 mm (**A, B**); 300 μm (**C**); 500 μm (**D**).

*Argopisteskeiseri* sp. nov. shows major similarities with *A.seyrigi* sp. nov. Both have the spur of hind tibiae distinctly elongate, extending significantly beyond the tibial apex (Figs 4B, 6B), black dorsal integuments (Figs 4A, 6A), and mostly confused elytral punctation. *Argopisteskeiseri* sp. nov. can be distinguished by the blackish abdomen and tibiae (mostly reddish brown in *A.seyrigi* sp. nov.) (Figs 4B, 6B). Both the median lobe of aedeagus and spermatheca are diagnostic for *Argopisteskeiseri* sp. nov.: median lobe (Fig. 4D) is thickset, with irregular outline in ventral view, and clearly sinuate in lateral view; spermatheca (Fig. 4C) has sinuate basal part and elongate, U-shaped, and uncoiled ductus.

##### Description of the holotype

(♂) . Body roundish in dorsal view (Fig. 4A), strongly convex in lateral view; total length of body (LB) = 3.48 mm; maximum pronotal width at the base (WP = 2.08 mm); maximum width of elytra in the middle (WE = 3.16 mm); WE/WP = 1.52. Dorsal integuments (Fig. 4A) entirely black with weak metallic reflections; ventral parts (Fig. 4B) entirely blackish; head black; frons and mouthparts black, with yellowish maxillary palpi; antennae yellowish (Fig. 4B); legs, including articulations, black, with yellowish tarsi (Fig. 4B). Head entirely hidden by the pronotum; vertex punctate, with a pair of large setiferous pores; area of frontal calli weakly raised; frons moderately elongate, roughly wrinkled; frontal ridge thin, weakly raised; frontogenal sutures thin, strongly raised; eyes large, elongate, slightly kidney-shaped; interantennal space clearly narrower than antennal sockets. Antennae (Fig. 4B) filiform, distinctly shorter than 1/2 the body length (LAN = 1.44 mm; LAN/LB = 0.41); segments 1–2 thicker; segments 3–11 slightly and gradually flattened; LA = 100:51:47:53:37:48:44:64:51:50:88. Pronotum (Fig. 4A) distinctly transverse (LP = 1.00 mm; WP/LP = 2.08); lateral margins strongly convergent anteriorly, straight, weakly expanded, not visible in dorsal view; basal margin arcuate and distinctly sinuate; surface sparsely micropunctate, with dense, small punctation; surface moderately raised parallel to the lateral margins; a large setiferous pore at the anterior angles. Scutellum small, subtriangular. Elytra (LE = 3.20 mm; LE/LP = 3.20) strongly curved laterally (Fig. 4A), approx. as long as wide (WE/LE = 1.02), jointly rounded apically; lateral margins finely bordered, visible in dorsal view; surface smooth; punctation small, dense, mostly confused, but arranged in some regular lines laterally, of which one is made of slightly larger punctures. Humeral calli moderately raised. Macropterous. Prosternum with posteriorly open procoxal cavities and large intercoxal prosternal process. Mesosternum very short. First abdominal sternite slightly longer than fifth (Fig. 4B); its central area bordered by ridges is quite wide, slightly narrowing posteriorly. Anterior and middle legs without modifications. Posterior femora greatly swollen (WF/LF = 0.68), elongate-subtriangular; posterior tibiae thick, distinctly shorter than femora, apically widened and prolonged into a spur-like process on inner side; outer side of hind tibia apically toothed; apical spur of hind tibiae simple, lanceolate, very elongate; first metatarsomere moderately enlarged. Median lobe of the aedeagus (LAED = 1.26 mm; LE/LAED = 2.54) (Fig. 4D) with smooth surface; in ventral view thickset, lanceolate but with slightly irregular outline; in lateral view median lobe moderately curved, thicker at the subapical part, with sinuate ventral outline; apex ventrally oriented; dorsal ligula formed by a central lobe medially incised in the apical part, and two thinner lateral lobes; its base at apical ~ 1/3.

##### Variability.

Male (*n* = 5; mean and standard deviation; range): LE = 3.08 ± 0.13 mm (2.88 ≤ LE ≤ 3.20 mm); WE = 2.98 ± 0.13 mm (2.82 ≤ WE ≤ 3.16 mm); LP = 0.92 ± 0.06 mm (0.86 ≤ LP ≤ 1.00 mm); WP = 1.98 ± 0.09 mm (1.88 ≤ WP ≤ 2.08 mm); LAN = 1.39 ± 0.15 mm (1.16 ≤ LAN ≤ 1.52 mm); LAED = 1.38 ± 0.09 mm (1.26 ≤ LAED ≤ 1.48 mm); LB = 3.37 ± 0.12 mm (3.20 ≤ LB ≤ 3.48 mm); LE/LP = 3.35 ± 0.11 (3.20 ≤ LE/LP ≤ 3.50); WE/WP = 1.50 ± 0.03 (1.44 ≤ WE/WP ≤ 1.53); WP/LP = 2.16 ± 0.04 (2.08 ≤ WP/LP ≤ 2.19); WE/LE = 0.97 ± 0.03 (0.94 ≤ WE/LE ≤ 0.99); LAN/LB = 0.41 ± 0.04 (0.35 ≤ LAN/LB ≤ 0.45); LE/LAED = 2.24 ± 0.18 (2.04 ≤ LE/LAED ≤ 2.54). Female (n = 11; mean and standard deviation; range): LE = 3.35 ± 0.04 mm (3.32 ≤ LE ≤ 3.40 mm); WE = 3.20 ± 0.06 mm (3.12 ≤ WE ≤ 3.24 mm); LP = 1.02 ± 0.03 mm (0.98 ≤ LP ≤ 1.04 mm); WP = 2.10 ± 0.05 mm (2.02 ≤ WP ≤ 2.12 mm); LAN = 1.34 ± 0.08 mm (1.28 ≤ LAN ≤ 1.44 mm); LSPC = 0.40 ± 0.01 mm (0.38 ≤ LSPC ≤ 0.40 mm); LB = 3.66 ± 0.07 mm (3.58 ≤ LB ≤ 3.72 mm); LE/LP = 3.30 ± 0.07 (3.23 ≤ LE/LP ≤ 3.39); WE/WP = 1.53 ± 0.01 (1.51 ≤ WE/WP ≤ 1.54); WP/LP = 2.06 ± 0.04 (2.04 ≤ WP/LP ≤ 2.12); WE/LE = 0.96 ± 0.02 (0.94 ≤ WE/LE ≤ 0.98); LAN/LB = 0.37 ± 0.01 (0.35 ≤ LAN/LB ≤ 0.39); LE/LSPC = 8.49 ± 0.19 (8.30 ≤ LE/LSPC ≤ 8.74).

Male and female paratypes very similar in shape, size, and color to the holotype. The arrangement of elytral punctation in 9 (+ 1 sutural) regular rows is better visible in some specimens. Spermatheca (Fig. 4C) with subcylindical, sinuate basal part; collum short; apical part moderately elongate, gradually narrowing, slightly wrinkled; ductus subventrally inserted, quite thickset, elongate, U-shaped, uncoiled.

##### Etymology.

The specific epithet refers to the first collector of the new species: Alfred “Fred” Kaiser (1895–1969) from Switzerland, renowned expert on DipteraSyrphidae from Madagascar.

##### Distribution.

Central-Eastern Madagascar (Toamasina province; Fig. 8A). Malagasy chorotype.

##### Ecological notes.

Host plant unknown. Collection localities fall within areas characterized by the vegetation division ‘Malagasy Evergreen & Semi-Evergreen Forest’.

#### ﻿ Argopistes laterosinuatus sp. nov.

Taxon classificationAnimaliaColeopteraChrysomelidae

B71ED5AF-E0BD-5908-B3DA-6D00048F45D6

https://zoobank.org/193ADA5E-1C06-4FB2-9337-AFC426E6D6A3

Figs 5 , 8A

##### Type material.

***Holotype*** ♀: “Coll. Mus. Congo / Madagascar: Antakotako / 15.i.1939, J. Vadon” [printed and handwritten on white card] [15°12.53'S; 49°47.61'E] (RMCA).

##### Diagnosis.

**Figure 5.**
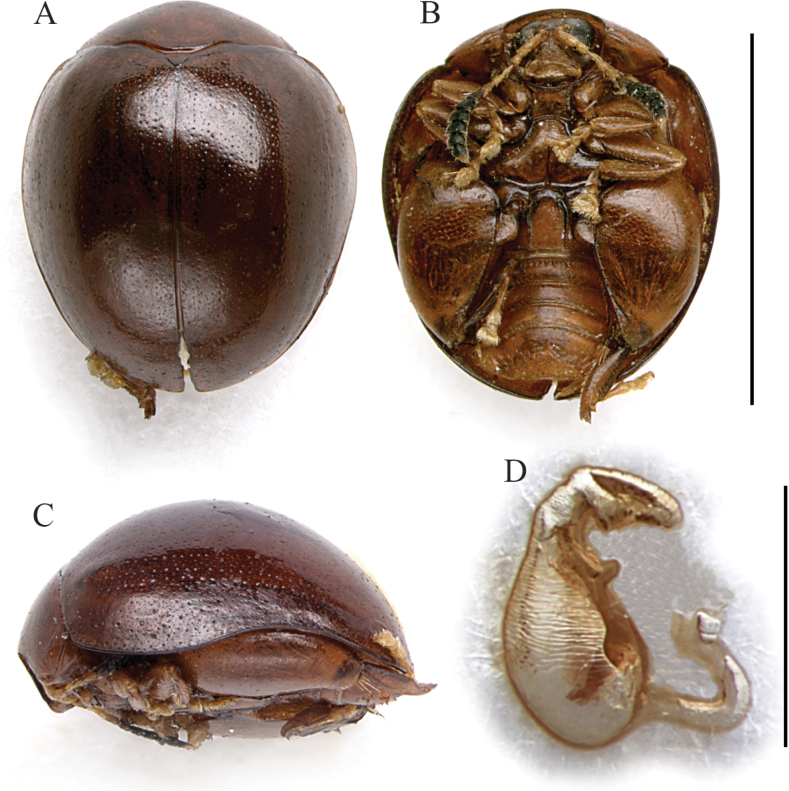
*Argopisteslaterosinuatus* sp. nov. **A** holotype, habitus in dorsal view **B** ibid, ventral view **C** ibid, lateral view **D** ibid, spermatheca. Scale bars: 3 mm (**A, B, C**); 300 μm (**D**).

*Argopisteslaterosinuatus* sp. nov. is easily recognizable among the Afrotropical *Argopistes* species due to its subovate outline in dorsal view (Fig. 5A) and sinuate sides in lateral view (Fig. 5C). Spermatheca is also diagnostic, due to the combination of pyriform basal part, with a distinct protrusion close to the collum, apical part clearly narrowing towards the apex, and ductus ventrally inserted, thickset, uncoiled (Fig. 5D).

##### Description of the holotype

(♀) . Body largely subovate in dorsal view (Fig. 5A), very convex in lateral view (Fig. 5C); total length of body (LB) = 3.18 mm; maximum pronotal width at the base (WP = 1.80 mm); maximum width of elytra at the basal third (WE = 2.72 mm); WE/WP = 1.51. Dorsal integuments (Fig. 5A) reddish brown with weak metallic reflections; ventral parts (Fig. 5B) light brown; head light brown; frons and mouthparts light brown; antennae (Fig. 5B) with segments 1–5 yellowish, 6 dark brown, 7–10 black, 11 black but distally lighter; legs entirely light brown (Fig. 5B). Head entirely hidden by the pronotum; vertex with small, irregular punctation, and a pair of large setiferous pores; frontal calli joined, moderately raised, with V-shaped posterior delimitation; frons moderately elongate, flat, roughly microreticulate; frontal ridge short; frontogenal sutures distinctly raised; eyes large, elongate, slightly kidney-shaped; interantennal space clearly narrower than antennal sockets. Antennae (Fig. 5B) clavate, slightly shorter than 1/2 the body length (LAN = 1.36 mm; LAN/LB = 0.43); LA = 100:36:53:47:48:52:44:46:49:52:79. Pronotum (Fig. 5A) strongly transverse (LP = 0.80 mm; WP/LP = 2.25); lateral margins strongly convergent anteriorly and slightly folded ventrally, weakly curved, weakly expanded, not visible in dorsal view; basal margin arcuate and distinctly sinuate; surface finely microreticulate, with very small and dense punctation; surface weakly raised near the lateral margins; a large setiferous pore at the anterior angles. Scutellum small, subtriangular. Elytra (LE = 2.92 mm; LE/LP = 3.65) slightly longer than wide (WE/LE = 0.93), strongly curved laterally in dorsal view (Fig. 5A) and distinctly sinuate in lateral view (Fig. 5C), jointly rounded apically; lateral margins finely bordered, visible in dorsal view; surface subsmooth, with very small and moderately dense, mostly confused punctation; slightly larger punctures are arranged in 9 (+ 1 sutural) regular rows. Humeral calli moderately raised. Macropterous. Prosternum with posteriorly open procoxal cavities and large intercoxal prosternal process. Mesosternum very short. First abdominal sternite approx. as long as fifth (Fig. 5B); its central area bordered by ridges is wide, rounded anteriorly, laterally subparallel. Anterior and middle legs without modifications. Posterior femora greatly swollen (WF/LF = 0.61), elongate-subtriangular; posterior tibiae thick, distinctly shorter than femora, apically widened and prolonged into a spur-like process on inner side; outer side of hind tibia apically toothed; apical spur of hind tibiae simple, lanceolate. Basal part of the spermatheca pyriform, with a distinct ventral protrusion close to the distal part (Fig. 5D); collum short, apical part short, narrowing towards the apex; ductus ventrally inserted, thickset, moderately elongate, uncoiled; LSPC = 0.32 mm; LE/LSPC = 9.13.

##### Variability.

Only the female holotype of the new species is known so far.

##### Etymology.

The specific epithet refers to the sinuate lateral margin of each elytra, a character absent in all other *Argopistes* species known to date for Madagascar.

##### Distribution.

North-eastern Madagascar (Toamasina province) (Fig. 8A). Malagasy chorotype.

##### Ecological notes.

Host plant unknown. The only known occurrence locality falls within an area characterized by the vegetation division ‘Malagasy Evergreen & Semi-Evergreen Forest’.

#### ﻿ Argopistes seyrigi sp. nov.

Taxon classificationAnimaliaColeopteraChrysomelidae

45D3FE60-EF95-573C-84D9-A54A264672AF

https://zoobank.org/62E50B82-6B80-4939-9127-07D64E0E9381

Figs 6 , 8A

##### Type material.

***Holotype*** ♂: “Coll. Mus Congo. / Madagascar: Mandraka / II.1944 / A. Seyrig” [printed on white card] [18°54.89'S; 47°55.61'E] (RMCA).

##### Diagnosis.

**Figure 6.**
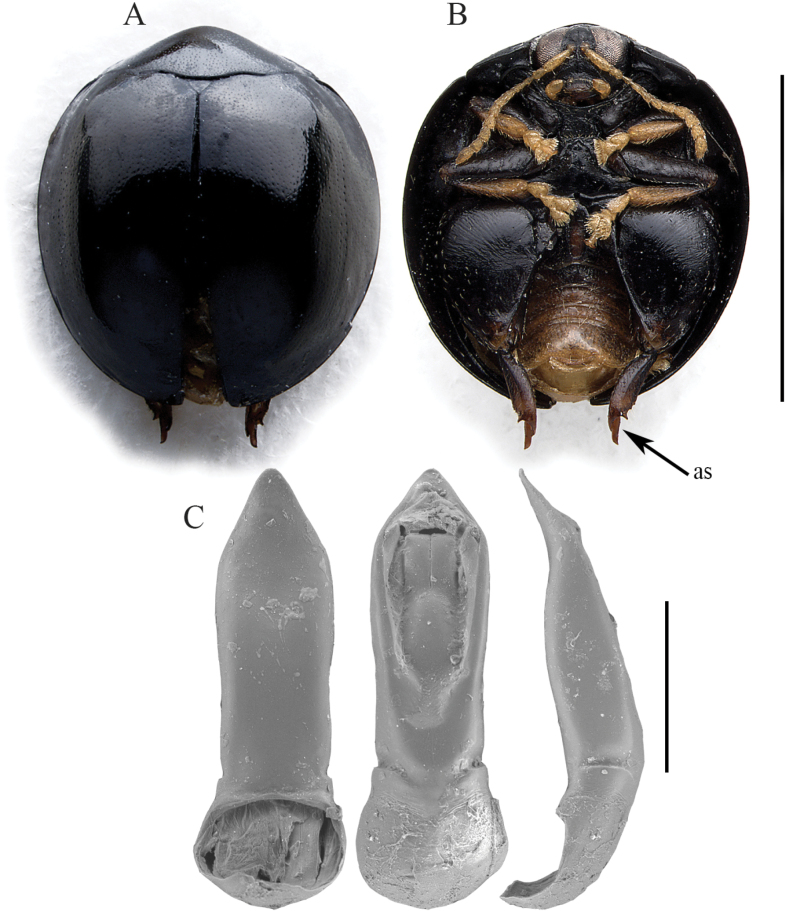
*Argopistesseyrigi* sp. nov. **A** holotype, habitus in dorsal view **B** ibid, ventral view **C** ibid, median lobe of the aedeagus. Abbreviations: as: apical spur of hind tibia. Scale bars: 3 mm (**A, B**); 500 μm (**C**).

Among the Malagasy *Argopistes* species, *A.seyrigi* sp. nov. shows strong similarities with *Argopisteskeiseri* sp. nov. Both have the spur of hind tibiae distinctly elongated, extending significantly beyond the tibial apex (Figs 4B, 6B), black dorsal integuments (Figs 4A, 6A), and mostly confused elytral punctation. *Argopistesseyrigi* sp. nov. can be distinguished by the mostly reddish brown abdomen and tibiae (blackish in *A.keiseri* sp. nov.) (Figs 4B, 6B). Median lobe of aedeagus of *A.seyrigi* sp. nov. has a clearly diagnostic value, due to the parallel sides in ventral view and the thinner apical part in lateral view (Fig. 6C).

##### Description of the holotype

(♂) . Body roundish in dorsal view (Fig. 6A), strongly convex in lateral view; total length of body (LB) = 3.52 mm; maximum pronotal width at the base (WP = 2.08 mm); maximum width of elytra at the middle (WE = 3.00 mm); WE/WP = 1.44. Dorsal integuments (Fig. 6A) entirely black with evident blueish metallic reflections; ventral parts (Fig. 6B) black, with mostly brownish abdomen; head black; frons and mouthparts black, with yellowish maxillary palpi; antennae yellowish (Fig. 6B); legs with black femora, hind tibiae dark brown, anterior and middle tibiae mostly light brown, and tarsi yellowish (Fig. 6B). Head entirely hidden by the pronotum; vertex punctate, with a pair of large setiferous pores; frontal calli joined, weakly delimited and weakly raised; frons short, roughly wrinkled; frontal ridge thin; frontogenal sutures quite thick and clearly raised; eyes large, elongate, slightly kidney-shaped; interantennal space clearly narrower than antennal sockets. Antennae (Fig. 6B) slightly shorter than 1/2 the body length (LAN = 1.44 mm; LAN/LB = 0.41), filiform; segments 1–2 thicker, segments 3–11 slightly and gradually flattened; LA = 100:46:37:44:40:39:41:43:43:38:75. Pronotum (Fig. 6A) clearly transverse (LP = 0.96 mm; WP/LP = 2.17); lateral margins strongly convergent anteriorly, straight, weakly expanded, not visible in dorsal view; basal margin arcuate and distinctly sinuate; surface microreticulate and micropunctate, with dense, small punctation; surface weakly raised parallel to the lateral margins; a large setiferous pore at the anterior angles. Scutellum small, subtriangular. Elytra (LE = 3.16 mm; LE/LP = 3.29) strongly curved laterally (Fig. 6A), approx. as long as wide (WE/LE = 0.95), jointly rounded apically; lateral margins finely bordered, visible in dorsal view; surface smooth; punctation very small, dense, less impressed than on pronotum, mostly confused, but arranged in some more impressed, barely visible regular lines, laterally. Humeral calli moderately raised. Macropterous. Prosternum with posteriorly open procoxal cavities and large intercoxal prosternal process. Mesosternum very short. First abdominal sternite approx. as long as fifth (Fig. 6B); its central area bordered by ridges is quite narrow, and slightly narrower posteriorly. Anterior and middle legs without modifications. Posterior femora greatly swollen (WF/LF = 0.69), elongate-subtriangular; posterior tibiae thick, distinctly shorter than femora, apically widened and prolonged into a spur-like process on inner side; outer side of hind tibia apically toothed; apical spur of hind tibiae simple, lanceolate, very elongate (Fig. 6B). Median lobe of the aedeagus (LAED = 1.36 mm; LE/LAED = 2.32) (Fig. 6C) with smooth surface; in ventral view lanceolate; in lateral view median lobe thicker in the middle part, moderately curved in the basal part, with sinuate ventral outline and straight apex; dorsal ligula formed by a central lobe, medially incised apically, and two thinner lateral lobes; its base at approx. the middle.

##### Variability.

Only the male holotype of the new species is known so far.

##### Etymology.

The specific epithet refers to the collector of the new species: André Seyrig (1897–1945) from France, an expert on Hymenoptera: Ichneumonidae, and a tireless collector of insects and plants in Madagascar.

##### Distribution.

Central-eastern Madagascar (Antananarivo province; Fig. 8A). Malagasy chorotype.

##### Ecological notes.

Host plant unknown. The only known occurrence locality falls within an area characterized by the vegetation division ‘Afromontane Moist Forest’.

#### ﻿ Argopistes vadoni sp. nov.

Taxon classificationAnimaliaColeopteraChrysomelidae

2F75BF1D-22A9-5822-AD38-430525759231

https://zoobank.org/F6866CF3-7109-43B3-8073-594223862553

Figs 7 , 8A

##### Type material.

***Holotype*** ♂: “Coll. Mus. Tervuren / N.E. Madagascar: / Ambodivoangy VII.1961/ J. Vadon” [printed on white card] [15°17.30'S; 49°36.88'E] (RMCA). Paratype ♀: “Coll. Mus. Congo / Madagascar: Antakotako / 15.i.1939 / J. Vadon” [printed on white card) [15°12.53'S; 49°47.61'E] (RMCA).

##### Diagnosis.

**Figure 7.**
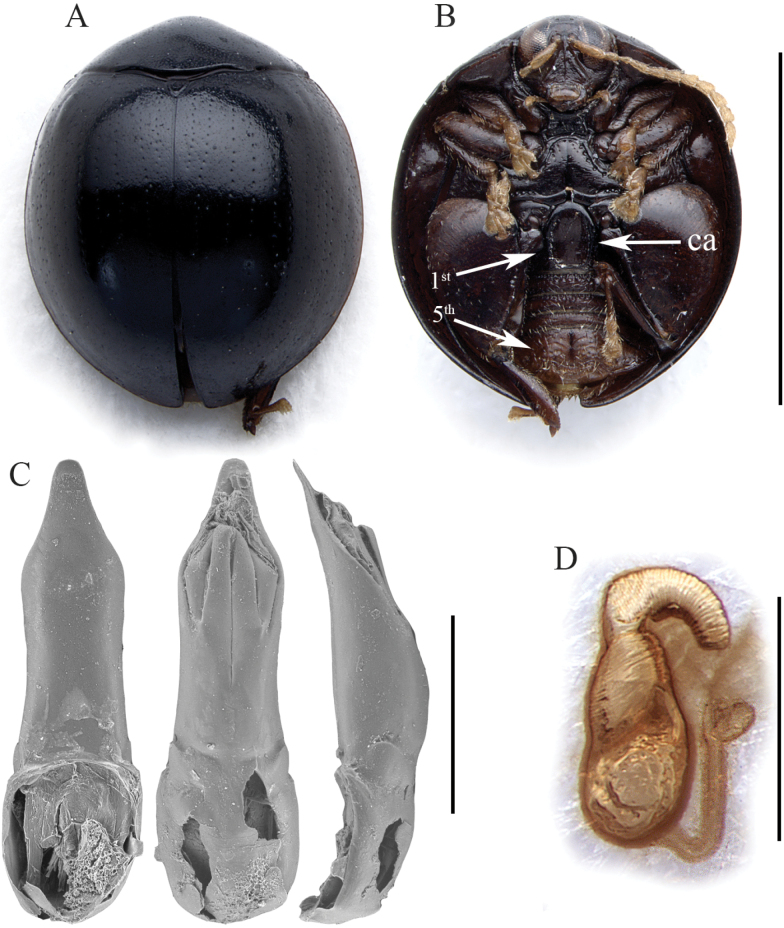
*Argopistesvadoni* sp. nov. **A** holotype, habitus in dorsal view **B** ibid, ventral view **C** ibid, median lobe of the aedeagus **D** spermatheca, from Antakotako. Abbreviations: 1^st^: first abdominal sternite; 5^th^: fifth abdominal sternite; ca: central area of the first abdominal sternite bordered by ridges. Scale bars: 3 mm (**A, B**); 500 μm (**C**); 300 μm (**D**).

*Argopistesvadoni* sp. nov. is one of the species with black or blackish dorsal integuments, and yellow and filiform antennae, but is distinguishable by the regular elytral punctation (Fig. 7A). Median lobe of the aedeagus and spermatheca are both diagnostic. Median lobe of the aedeagus is easily recognizable by the apical part, distinctly slender in ventral view (Fig. 7C). Spermatheca is unique for the combination of pyriform basal part, distal part homogenously thickened, and ductus subapically inserted, quite thickset, moderately elongated, and uncoiled (Fig. 7D).

##### Description of the holotype

(♂) . Body roundish in dorsal view (Fig. 7A), strongly convex in lateral view; total length of body (LB) = 3.32 mm; maximum pronotal width at the base (WP = 2.00 mm); maximum width of elytra in the middle (WE = 2.84 mm); WE/WP = 1.42. Dorsal integuments (Fig. 7A) entirely black with weak blueish metallic reflections; ventral parts (Fig. 7B) dark reddish brown; head dark brown; frons and mouthparts brown, with yellowish maxillary palpi; antennae (Fig. 7B) yellowish; legs, including articulations, reddish brown, with yellowish tarsi (Fig. 7B). Head entirely hidden by the pronotum; vertex punctate, with a pair of large setiferous pores; frontal calli joined, clearly delimited and straight posteriorly; frons elongate, flat, roughly wrinkled; frontal ridge elongate, thin and sharp; frontogenal sutures distinctly raised; eyes large, elongate, slightly kidney-shaped; interantennal space clearly narrower than antennal sockets. Antennae (Fig. 7B) filiform, as long as ~ 1/2 the body length (LAN = 1.76 mm; LAN/LB = 0.53); segments 1 and 2 thicker; segments 3–11 slightly and gradually flattened; LA = 100:42:33:47:47:40:41:44:43:39:64. Pronotum (Fig. 7A) distinctly transverse (LP = 0.96 mm; WP/LP = 2.08); lateral margins strongly convergent anteriorly, weakly curved, weakly expanded, not visible in dorsal view; basal margin arcuate and distinctly sinuate; surface finely wrinkled, with very dense, small punctation; surface weakly raised parallel to the lateral margins; a large setiferous pore at the anterior angles. Scutellum small, subtriangular. Elytra (LE = 2.98 mm; LE/LP = 3.10) strongly curved laterally (Fig. 7A), approx. as long as wide (WE/LE = 0.95), jointly rounded apically; lateral margins finely bordered, visible in dorsal view; surface micropunctate; main punctation small, arranged in 9 (+ 1 sutural) regular rows, more confused along lateral parts. Humeral calli moderately raised. Macropterous. Prosternum with posteriorly open procoxal cavities and large intercoxal prosternal process. Mesosternum very short. First abdominal sternite (Fig. 7B) slightly longer than fifth; its central area bordered by ridges is wide, subovate. Anterior and middle legs without modifications. Posterior femora greatly swollen (WF/LF = 0.67), elongate-subtriangular; posterior tibiae thick, distinctly shorter than femora, apically widened and prolonged into a spur-like process on inner side; outer side of hind tibia apically toothed; apical spur of hind tibiae simple, lanceolate; first metatarsomere moderately enlarged. Median lobe of the aedeagus (LAED = 1.24 mm; LE/LAED = 2.40) (Fig. 7C) with smooth surface; widest at the basal opening in ventral view, slightly curved inwardly; distal part distinctly thinner, sides convergent towards the rounded apex; in lateral view median lobe weakly curved, thicker in the central third; dorsal ligula formed by a central lobe, medially incised, and two lateral lobes; its base at apical ~ 1/3.

##### Variability.

Female paratype very similar in shape and color to the holotype. LE = 3.40 mm; WE = 3.16 mm; LP = 1.00 mm; WP = 2.12 mm; LAN = 1.72 mm; LSPC = 0.34 mm; LB = 3.60 mm; LE/LP = 3.40; WE/WP = 1.49; WP/LP = 2.12; WE/LE = 0.93; LAN/LB = 0.48; LE/LSPC = 10.00. First metatarsomere in female not enlarged. Spermatheca (Fig. 7D) apparently wrinkled on most surface; basal part pyriform; collum short; distal part moderately elongate, apically truncate; ductus subapically inserted, quite thickset, moderately elongate, uncoiled.

##### Etymology.

The specific epithet refers to the collector of the new species: Jean Vadon (1904–1970) from France, one of the fathers of the entomological research in Madagascar.

##### Distribution.

**Figure 8.**
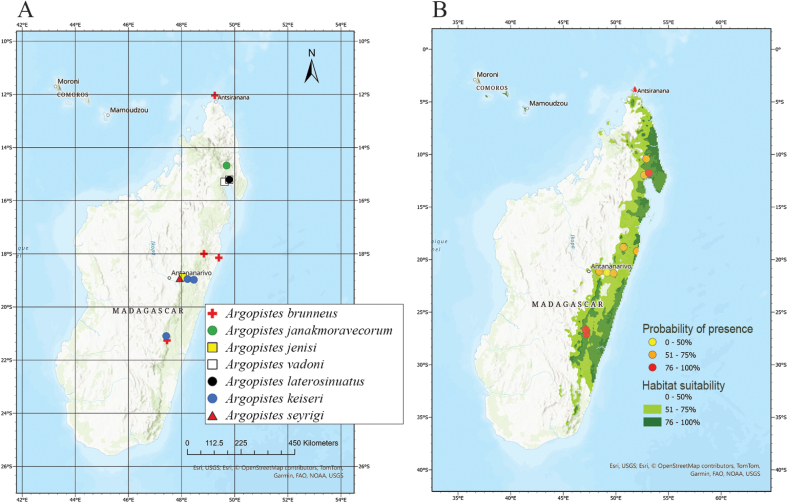
Distribution and habitat suitability of the *Argopistes* species in Madagascar. **A** occurrence locality for each species **B** probability of presence of the occurrence localities, and potential suitability areas for the genus in Madagascar from ENMs.

Northern-eastern Madagascar (Toamasina province; Fig. 8A). Malagasy chorotype.

##### Ecological notes.

Host plant unknown. The two known occurrence localities fall within areas characterized by the vegetation division ‘Malagasy Evergreen & Semi-Evergreen Forest’.

### ﻿Key to species

**Table:** 

1	Antennae clavate, with segments 6–11 clearly dilated and strongly blackened (Figs 2B, 5B)	**2**
–	Antennae filiform, at most with gradually enlarged distal segments, entirely yellowish (Figs 1B, 3B, 4B, 6B, 7B)	**3**
2	Body shape roundish. Dorsal integuments black. Elytral sides not sinuate in lateral view. Elytral punctation with dense and small punctures on the disc, without evident regular rows (Fig. 2A). Spermatheca with subpyriform basal part; ductus elongate, distally clearly coiled (Fig. 2C). Male unknown	***Argopistesjanakmoravecorum* sp. nov.** (Figs 2, 8A)
–	Body shape subovate, more elongate. Dorsal integuments reddish brown. Elytral sides distinctly sinuate in lateral view (Fig. 5C). Elytral punctation with sparser but larger punctures on the disc, with hints of 9 (+ 1 sutural) regular rows (Fig. 5A, C). Spermatheca with pyriform basal part; ductus shorter, uncoiled (Fig. 5D). Male unknown	***Argopisteslaterosinuatus* sp. nov.** (Figs 5, 8A)
3	Apical spur of hind tibiae distinctly elongate, extending significantly beyond the tibial apex (as in Figs 4, 6)	**4**
–	Apical spur of hind tibiae shortly extending beyond the tibial apex (as in Fig. 1)	**5**
4	Dorsal integuments intense black with weak metallic reflections; abdomen and tibiae blackish (Fig. 4B). Median lobe of the aedeagus thickset, clearly sinuate in lateral view (Fig. 4D). Spermatheca with subcylindrical, sinuate basal part; ductus elongate, subapically oriented, uncoiled (Fig. 4C)	***Argopisteskeiseri* sp. nov.** (Figs 4, 8A)
–	Dorsal integuments black but with clear blueish metallic reflections; abdomen and tibiae mostly reddish brown (Fig. 6B). Median lobe of aedeagus slender, in lateral view with narrow and straight apical part (Fig. 6C). Female unknown	***Argopistesseyrigi* sp. nov.** (Figs 6, 8A)
5	Elytra in dorsal view with slightly parallel lateral margins (Fig. 1A, B). Central area of the first abdominal sternite bordered by ridges is narrow, subrhomboidal (ca: Fig. 1B). Dorsal integuments variable, black to at least partially reddish brown, especially on pronotum; sometimes elytra black with brown elytral patches (Fig. 1A, C). Median lobe of the aedeagus tapered towards the apex in ventral view (Fig. 1D). Spermatheca with subcylindrical, dorsally enlarged basal part; ductus thin, short, subapically oriented (Fig. 1E)	***Argopistesbrunneus* Weise** (Figs 1, 8A)
–	Elytra in dorsal view, with clearly rounded lateral margins (Figs 3A, B, 7A, B). Central area of the first abdominal sternite bordered by ridges is wide, laterally subparallel (ca: Figs 3A, 7A). Dorsal integuments entirely black (Figs 3B, 7B). Median lobe of the aedeagus and/or spermatheca differently shaped	**6**
6	Elytral punctation small, dense, almost completely unordered (Fig. 3A). Dorsal integuments, ventral integuments, and legs intense black with evident blueish metallic reflections, except the yellowish tarsi (Fig. 3A, B). First abdominal sternite distinctly shorter than fifth (Fig. 3B). Basal part of the spermatheca subglobose, ventrally enlarged; distal part gradually narrowed apically (Fig. 3C). Male unknown	***Argopistesjenisi* sp. nov.** (Figs 3, 8A)
–	Elytral punctation with larger punctures ordered in regular rows (Fig. 7A). Dorsal integuments black; ventral integuments and legs brownish, except the yellowish tarsi (Fig. 7B). First abdominal sternite distinctly longer than fifth (Fig. 7B). Spermatheca (Fig. 7D) with pyriform basal part; distal part not narrowed apically. Apical part of the median lobe of aedeagus distinctly slender in ventral view (Fig. 7C)	***Argopistesvadoni* sp. nov.** (Figs 7, 8A)

### ﻿Habitat suitability

VIF and Pearson’s correlation analyses returned a set of nine uncorrelated bioclimatic variables which were then used to calibrate the models: BIO2, BIO3, BIO8, BIO9, BIO13, BIO14, BIO15, BIO18, and BIO19. The ensemble models for the genus *Argopistes* (Fig. 8) resulted in high performance scores (AUC = 0.899 and CBI = 0.754), indicating a continuous area of habitat suitability in the Eastern part of Madagascar, a region characterized in particular by vegetation formations (cf. Sayre et al. 2013) such as the ‘Tropical Lowland Humid Forest’ in the central area, mainly with the ‘Malagasy Evergreen & Semi-Evergreen Forest’ division, and to a lesser extent the ‘Tropical Seasonally Dry Forest’ to the north, with the ‘Malagasy Dry Deciduous and Evergreen Forest and Woodland’ division. Based on our model, in Madagascar the areas with high habitat suitability for the genus *Argopistes* are characterized by: a) mean diurnal range temperature (BIO2) with values between 5 and 10 °C; b) mean temperature of wettest quarter (BIO8) between 12 and 24 °C; c) precipitation of wettest month (BIO13) not exceeding 500 mm; d) coefficient of variation (BIO15), as a measure of precipitation inter-annual variability, lower than 30%. Therefore, the western part of Madagascar does not offer optimal conditions for the occurrence of *Argopistes* species (Fig. 8B).

## ﻿Discussion

Based on our revision, *Argopistes* is present in Madagascar with seven endemic species.

The six new *Argopistes* species here described unequivocally display the typical characters of the genus (Biondi and D’Alessandro 2012; Nadein 2015): body ovate to rounded in dorsal view, strongly convex in lateral view; head generally entirely hidden by the pronotum; antennae short, their length not exceeding 1/2 of the body length; head opisthognathous; eyes very large, kidney-shaped; antennal sockets very close to each other, their distance generally shorter than their diameter; frontogenal sutures (edges of antennal grooves) distinctly raised, often more evident than frontal ridge; frontal calli medially jointed, usually distinctly delimited from vertex; vertex with a pair of large setiferous pores; pronotum always covered with punctures, without evident grooves and impressions; posterior edge clearly bisinuate; scutellum visible, subtriangular; elytra glabrous, with small punctation, confused or arranged in regular striae; epipleura orientation subvertical; prosternum with posteriorly open procoxal cavities and large intercoxal prosternal process; mesosternum very short; central area of the first abdominal sternite bordered by ridges; anterior and middle legs without special modifications; posterior femora considerably swollen, elongate-subtriangular; posterior tibiae thick, short, apically widened and prolonged into a spur-like process on inner side; outer side of hind tibia apically toothed; apical spur of hind tibiae simple, evident. Based on the general body shape, *Argopistes* species are apparently very similar to Coccinellidae, especially of the genus *Exochomus* Redtenbacher, as Motschulsky (1860) highlighted.

*Argopistesjanakmoravecorum* sp. nov. and *A.laterosinuatus* sp. nov. show clear similarities based on the antennal and spermathecal morphology (Figs 2B, C, 5B, D). Some of the spermathecal characters, such as the general shape of the distal part and a more or less evident, apparently cup-shaped formation on the basal part (Figs 2C, 5D), are present in other *Argopistes* species, also outside the Afrotropical region (Blanco and Konstantinov 2013). However, the clavate and blackened antennae are unique, making the two species taxonomically isolated from the remaining Malagasy and sub-Saharan *Argopistes* species.

Based on the available ecological data, Asian and New World *Argopistes* species and many Afrotropical species are associated with Oleaceae (Blanco and Konstantinov 2013). In sub-Saharan Africa, *Argopistes* species are primarily associated with Olive trees [Oleaeuropaeavar.africana (Mill.)], on which larvae are leaf miners, and adults are defoliators (Biondi and D’Alessandro 2012; Hlaka et al. 2022). No ecological data are available for the Malagasy species. However, based on our habitat suitability model, the western part of Madagascar does not offer optimal conditions for their occurrence (Fig. 8B). Indeed, Malagasy species are distributed in the central and eastern areas of the Island (Fig. 8A). It must be emphasized that Madagascar has been interested in a significant loss of natural habitats over decades so that species described on preserved specimens collected a long time ago might have become rare or even locally extinct (Goodman 2022). That makes it crucial to document Malagasy biodiversity as soon as possible and check its status through field campaigns. Following that principle, in this contribution, we described new species even on single males or females, being confident in the reliability of the diagnostic value of the illustrated characters.

## Supplementary Material

XML Treatment for Argopistes brunneus

XML Treatment for Argopistes janakmoravecorum

XML Treatment for Argopistes jenisi

XML Treatment for Argopistes keiseri

XML Treatment for Argopistes laterosinuatus

XML Treatment for Argopistes seyrigi

XML Treatment for Argopistes vadoni

## ﻿Additional information

### Author ORCIDs

Maurizio Biondi https://orcid.org/0000-0002-4481-9152

Mattia Iannella https://orcid.org/0000-0003-4695-0194

Paola D'Alessandro https://orcid.org/0000-0002-4481-9152

### Data availability

All of the data that support the findings of this study are available in the main text.
